# Impact of smoking status on Sjögren’s disease diagnosis and phenotype: results from a cohort study with matched population controls

**DOI:** 10.1093/rheumatology/keag334

**Published:** 2026-06-24

**Authors:** Matilde Bandeira, Aliaksandra Baranskaya, Valentina Pucino, Saba Nayar, Jon Higham, Ana Poveda-Gallego, Saaeha Rauz, Simon J Bowman, Benjamin A Fisher

**Affiliations:** Rheumatology Department, Unidade Local de Saúde Santa Maria, Centro Académico de Medicina de Lisboa (CAML), Lisbon, Portugal; Rheumatology Department, Unidade Local de Saúde Viseu Dão Lafões, Viseu, Portugal; Faculdade de Medicina, Universidade de Lisboa, CAML, Lisbon, Portugal; School of Infection, Inflammation and Immunology, University of Birmingham, Birmingham, UK; School of Infection, Inflammation and Immunology, University of Birmingham, Birmingham, UK; Department of Rheumatology, Queen Elizabeth Hospital, University Hospitals Birmingham NHS Foundation Trust, Birmingham, UK; National Institute for Health and Care Research (NIHR) Birmingham Biomedical Research Centre, University Hospitals Birmingham NHS Foundation Trust, Birmingham, UK; School of Infection, Inflammation and Immunology, University of Birmingham, Birmingham, UK; Department of Rheumatology, Queen Elizabeth Hospital, University Hospitals Birmingham NHS Foundation Trust, Birmingham, UK; Department of Clinical and Experimental Medicine, University of Pisa, Pisa, Italy; School of Infection, Inflammation and Immunology, University of Birmingham, Birmingham, UK; National Institute for Health and Care Research (NIHR) Birmingham Biomedical Research Centre, University Hospitals Birmingham NHS Foundation Trust, Birmingham, UK; Department of Oral Medicine, Birmingham Dental Hospital, Birmingham, UK; Department of Oral Medicine, Birmingham Dental Hospital, Birmingham, UK; School of Infection, Inflammation and Immunology, University of Birmingham, Birmingham, UK; National Institute for Health and Care Research (NIHR) Birmingham Biomedical Research Centre, University Hospitals Birmingham NHS Foundation Trust, Birmingham, UK; Academic Unit of Ophthalmology, Birmingham and Midland Eye Centre, Sandwell and West Birmingham NHS Trust, Birmingham, UK; School of Infection, Inflammation and Immunology, University of Birmingham, Birmingham, UK; Department of Rheumatology, Queen Elizabeth Hospital, University Hospitals Birmingham NHS Foundation Trust, Birmingham, UK; National Institute for Health and Care Research (NIHR) Birmingham Biomedical Research Centre, University Hospitals Birmingham NHS Foundation Trust, Birmingham, UK; School of Infection, Inflammation and Immunology, University of Birmingham, Birmingham, UK; Department of Rheumatology, Queen Elizabeth Hospital, University Hospitals Birmingham NHS Foundation Trust, Birmingham, UK; National Institute for Health and Care Research (NIHR) Birmingham Biomedical Research Centre, University Hospitals Birmingham NHS Foundation Trust, Birmingham, UK

**Keywords:** Sjögren’s disease, smoking, risk factors, tobacco, sicca, healthy controls

## Abstract

**Objectives:**

To examine associations between smoking and Sjögren’s disease (SjD) diagnosis, compared with non-Sjögren’s sicca syndrome (sicca) and population controls, and to explore immunomodulatory effects of smoking on disease phenotype.

**Methods:**

Adults referred for suspected SjD to the Optimising Assessment in Sjögren’s Syndrome (OASIS) cohort from 2014 until 2023 were classified as SjD or sicca. Smoking status, exposure (pack-years) and passive smoking history were recorded through standardized questionnaires. Associations with diagnosis, clinical and serological parameters, and patient-reported outcomes were analysed using multivariate logistic and linear regression. Smoking patterns were further compared with matched controls from the 2018 UK Annual Population Survey.

**Results:**

A total of 317 SjD patients and 170 sicca controls were included. Of these, 257 SjD met 2016 classification criteria, and were used for primary analyses. Smoking data were available for 85.0% of the cohort. SjD patients were more frequently never smokers than sicca (70.5% *vs* 53.1%, *P = *0.002). In the multivariate analysis, current smoking (odds ratio 0.37, 95% CI 0.15–0.92) was independently associated with lower odds of SjD. Never smokers exhibited higher IgG levels (*P = *0.003) and more frequent biological EULAR Sjögren’s Disease Activity Index activity (*P = *0.038). Smoking exposure correlated with reduced IgG, RF levels and seropositivity, independent of DMARD use. Compared with population controls, sicca, but not SjD, showed higher ever-smoking rates (49.6% *vs* 39.1%, *P = *0.049).

**Conclusion:**

Smoking was negatively associated with SjD diagnosis compared with sicca, but not population controls. Smoking exposure was linked to lower immunoglobulin and autoantibody levels, suggesting an immunomodulatory effect rather than true disease protection. Findings support a potential metabolically influenced, non-autoimmune, sicca phenotype linked to smoking.

## Graphical abstract

**Graphical Abstract:**
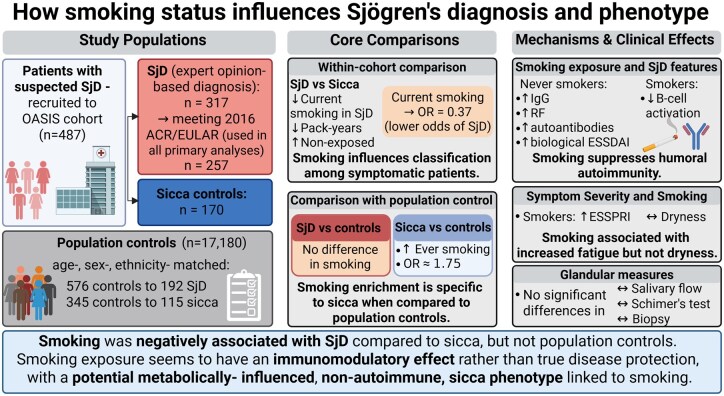

Rheumatology key messages

- Smoking is inversely associated with Sjögren’s disease compared with sicca, but not to population controls.
- Smoking exposure links to lower immunoglobulin and autoantibody levels, suggesting immunomodulatory effects.
- Higher smoking prevalence in sicca individuals supports a metabolically influenced, non-autoimmune phenotype.

## Introduction

Sjogren’s disease (SjD) is an autoimmune disease characterized by mucosal dryness with a proportion of patients also having extra-glandular systemic features. More than 20 genetic risk alleles have been identified in genome-wide association studies [1], but familial transmission (heritability and shared environment) has been estimated at 0.54 [2]. However, little is known about environmental risk factors. Smoking is positively associated with several autoimmune diseases such as RA but negatively associated with others. A few studies have investigated the relationship between SjD and smoking but with ambiguous conclusions.

The majority of the literature points to a negative correlation between active smoking and SjD diagnosis [3–10]. In fact, a recent meta-analysis including five studies demonstrated that current smoking is inversely related to SjD [pooled odds ratio (OR) 0.49, 95% CI 0.29–0.83, *P = *0.008] [11].

The impact of past smoking is less clear [11], with some studies indicating it could be a risk factor for developing the disease and others showing the same negative correlation as current smoking. Reasons for these divergent findings include differences in methodology, sample size, SjD recruitment and case definition, and control population. The latter is particularly important given that there seems to be a tendency for a positive association between past smoking and SjD when comparing them with healthy controls, and a negative association when comparing SjD with non-Sjögren’s sicca syndrome (sicca) controls [12]. Given that sicca could be considered a disease control group, it is difficult to know whether differences in smoking between these populations reflects less smoking in SjD or more in sicca.

To date, only one study has explored the relationship between passive smoking and risk of SjD. Karabulut *et al*. [3] found that, compared with healthy controls, SjD patients were less frequently current smokers and had shorter duration of smoking as well as lower pack-year units, but were more frequently past, ever and passive smokers.

The reasons behind the frequently observed negative association between smoking and SjD are unclear. Some data suggest that smoking is linked to reduced salivary gland inflammation [12]. Another possibility is that smoking affects the phenotype of an evolving CTD, similar to the opposing association of smoking observed with Crohn’s and ulcerative colitis. The possibility of reverse causation, where patients stop smoking because of early dryness symptoms, has also not been ruled out.

We set out to investigate the association between smoking and SjD diagnosis and disease activity by using a well-characterized cohort of newly presenting SjD patients with detailed smoking data and comparing with sicca from the same cohort, and with matched population-based controls.

## Methods

### Patient recruitment and inclusion criteria

Patients referred because of suspected SjD were recruited into the Optimising Assessment in Sjögren’s Syndrome (OASIS) cohort established in Birmingham, UK (2014–23). Data from all patients included up to October 2023 was obtained. The OASIS cohort includes consecutively referred patients with suspected SjD, some of whom have an established diagnosis at the time of inclusion. At enrolment into OASIS, demographic data, symptom duration and disease activity scores, including the EULAR Sjögren’s Disease Activity Index (ESSDAI), are collected. Tear production is assessed by Schirmer’s test without anaesthetic, and unstimulated salivary flow over 5 min is measured. Subjects are also given questionnaires that include several patient-reported outcomes, such as the EULAR Sjögren’s Syndrome Patient Reported Index (ESSPRI), and questions on hypothetical risk factors. All previous diagnoses are re-evaluated, but, when confirmed, the original date of diagnosis is retained.

For the primary analysis we used patients who met 2016 ACR/EULAR classification criteria in the absence of another CTD. Sicca controls were only included if all of the following criteria were met: they did not have a physician diagnosis of SjD, did not meet 2016 classification criteria, were anti-Ro antibody negative and had no obvious systemic inflammatory cause for their symptoms. For a secondary analysis we defined SjD as all patients with an expert opinion-based physician diagnosis of SjD regardless of whether they fulfilled classification criteria.

### Smoking data collection

Smoking status was regularly obtained within clinic with additional information available through this risk factor questionnaire. Patients were divided into current smokers, past smokers and never smokers based on smoking status questions available. Cigarette consumption, defined as pack-years, was also calculated based on the questionnaire detailing date when smoking started and stopped, if applicable, as well as average amount per day. Information on passive smoking was collected with the questions ‘Have you ever lived with anybody else who smoked inside the house?’ and ‘Have you ever worked in an environment where someone regularly smoked inside your workplace?’ Integrating this information, patients were also divided into non-exposed, i.e. never smokers with no passive smoking history, exclusively passive smokers and ever smokers (current and past smokers combined).

### Healthy controls data

Annual Population Survey (APS) data from January–December 2018 [13], based on the median year of recruitment for OASIS, were used for population control and case matching analysis. APS is an annually run survey of persons living in the UK covering topics such as health, education, ethnicity and employment. Data from this survey are used by the Office of National Statistics to report on adult smoking habits [14]. Controls aged ≥18 years, residing in West Midlands (similar to the majority of the OASIS cohort), with available smoking data, were considered, with a final number used of 17 180 out of the total 284 104.

Smoking data in APS is collected using the question ‘Have you ever smoked cigarettes regularly?’ with a further sub-question on current cigarette smoking status. From these two variables, individuals were categorized as ever/never smoker and current/past/never smoker.

### Statistical analysis

Continuous variables were presented as median or mean ± s.d., according to distribution. Categorical variables presented as relative frequencies. Frequencies of categorical variables were compared using χ^2^ or Fisher’s test, whereas continuous variables were compared using Student’s *t*-test or Mann–Whitney Test, as appropriate (according to normality and variance homogeneity).

Correlation between continuous variables was tested with Pearson or Spearman, according to their distribution. Predictors of SjD diagnosis were identified through binomial logistic regression modelling, as well as linear regression modelling for IgG levels, after appropriate collinearity analysis.

For the healthy controls, matching was performed separately for sicca and SjD patients with a 3:1 ratio (controls:cases) to maximize statistical power, using ‘FUZZY’ matching, a nearest-neighbour approach allowing prespecified tolerance thresholds for some variables while requiring exact matches for others. Matching variables were sex (female *vs* male, 0 tolerance), ethnicity (White *vs* non-White, 0 tolerance), available smoking data (0 tolerance) and age (1 year tolerance).

In addition to matched analyses, binary logistic regression models were generated using the full APS control dataset (*n* = 17180). Univariate and multivariate models were performed using two smoking categorizations: (i) ever/never and (ii) current/past/never smokers, with multivariate models adjusted for age, sex and ethnicity (White *vs* non-White). Complete case approach was used in the multivariate regression models.

Statistical significance was set at *P < *0.05. All statistical analysis was performed with SPSS/IBM version 29.0.1.

### Ethical considerations

This study has been conducted according to the principles of the Declaration of Helsinki and was approved by the Wales Research Ethics Committee 7 (WREC 7) formerly Dyfed Powys REC; 13/WA/0392. All patients have provided signed informed consent. All data were pseudo-anonymized. APS data was accessed by A.B. as per the terms and conditions described in the End User Licence Agreement from UK Data Service.

## Results

A total of 487 OASIS cohort patients were included in this study, with 170 individuals diagnosed with sicca and 317 with a clinical diagnosis of SjD. Of those diagnosed with SjD, 81.1% (*n* = 257) met the 2016 Sjögren’s ACR/EULAR classification criteria. Smoking status information was available for 85.0% (*n* = 414) of the total cohort.

### Smoking status and SjD diagnosis

Compared with sicca (Table 1), those diagnosed with SjD and meeting the 2016 ACR/EULAR classification criteria were more frequently never smokers [70.5% (*n* = 155/220) *vs* 53.1% (*n* = 76/143), *P = *0.002] and less frequently current [5.9% (*n* = 13/220) *vs* 12.6% (*n* = 18/143), *P = *0.002] and past [23.6% (*n* = 52/220) *vs* 34.3% (*n* = 49/143), *P = *0.002] smokers. (Table 1).

**Table 1:** Clinical and laboratorial characteristics in Sjögren’s disease and sicca.

			Sjögren’s disease meeting 2016 CC (*n* = 257)	Sicca (*n* = 169)	Univariate analysis (*P*-value)
Age at inclusion, years			54.0 ± 15.2 (256)	54.9 ± 12.0 (167)	0.555
Age at diagnosis, years			49.5 ± 15.5 (238)		
Age at symptom onset, years			45.7 ± 15.5 (248)	48.0 ± 13.3 (155)	0.131
Symptom duration, years			8.4 ± 8.9 (249)	6.9 ± 7.2 (157)	0.078
Female sex			242/257 (94.2)	151/169 (89.3)	0.094
**Ethnicity**	**White**		**134/205 (65.4)**	**104/118 (88.1)**	**<0.001**
	**Asian**		**50/205 (24.4)**	**10/118 (8.5)**	
	**Black**		**9/205 (4.4)**	**3/118 (2.5)**	
	**Other**		**12/205 (5.9)**	**1/118 (0.8)**	
BMI, kg/m^2^			27.4 ± 9.0 (249)	28.5 ± 6.6 (163)	0.188
Patients who fulfil classification criteria					
** 2002 AECG CC**			**247/257 (96.1)**	**0/165 (0.0)**	**<0.001**
** 2016 ACR/EULAR CC**			**257/257 (100.0)**	**0/165 (0.0)**	**<0.001**
Clinical data					
Smoking					
** Smoking status**	**Never smoker**		**155/220 (70.5)**	**76/143 (53.1)**	**0.002**
	**Current smoker**		**13/220 (5.9)**	**18/143 (12.6)**	
	**Past smoker**		**52/220 (23.6)**	**49/143 (34.3)**	
** Smoking status**	**Non exposed**		**49/157 (31.2)**	**24/118 (20.3)**	**0.032**
	**Passive smoker**		**43/157 (27.4)**	**27/118 (22.9)**	
	**Ever smoker**		**65/157 (41.4)**	**67/118 (56.8)**	
** Pack-year units**			**10.8 ± 11.0 (37)**	**15.3 ± 13.1 (40)**	**0.048**
Regular passive smoking in all	Home		74/144 (51.4)	64/110 (58.2)	0.310
	Quantity at home	<10 cigarettes/day	25/71 (35.2)	17/61 (27.9)	0.454
		≥10 cigarettes/day	46/71 (64.8)	44/61 (72.1)	
	Work		46/153 (30.1)	42/111 (37.8)	0.190
	Anywhere		80/141 (56.7)	72/108 (66.7)	0.118
Regular passive smoking in never smokers only	Home		39/95 (41.1)	24/53 (45.3)	0.729
	Quantity at home	<10 cigarettes/day	17/36 (47.2)	9/21 (42.9)	0.789
		≥10 cigarettes/day	19/36 (52.8)	12/21 (57.1)	
	Work		24/104 (23.1)	13/54 (24.1)	1.000
	Anywhere		43/92 (46.7)	27/51 (52.9)	0.491
Smoking cessation	>5 years before symptom onset		28/39 (71.8)	25/37 (67.6)	0.725
	<5 years before symptom onset		7/39 (17.9)	5/37 (13.5)	
	<5 years after symptom onset		2/39 (5.1)	4/37 (10.8)	
	>5 years after symptom onset		2/39 (5.1)	3/37 (8.1)	
Δ between smoking cessation and symptom onset, years			15.9 ± 11.1 (39)	13.7 ± 10.1 (37)	0.374
Alcohol	No alcohol consumption		81/184 (44.0)	41/133 (30.8)	0.058
	Social drinker only		40/184 (21.7)	35/133 (26.3)	
	Regular consumption		63/184 (34.2)	57/133 (42.9)	
** Schirmer’s test ≤5 mm/5 min**			**160/244 (65.6)**	**69/161 (42.9)**	**<0.001**
** Unstimulated salivary flow ≤0.1 mL/min**			**155/242 (64.0)**	**82/162 (50.6)**	**0.008**
** Unstimulated salivary flow, mL/min**			**0.1 ± 0.2 (241)**	**0.2 ± 0.3 (159)**	**<0.001**
Dental caries			58/219 (26.5)	27/147 (18.4)	0.078
** Tooth loss**			**116/222 (52.3)**	**60/145 (41.4)**	**0.043**
**Positive anti-SSA**			**226/257 (87.9)**	**0/165 (0.0)**	**<0.001**
**Positive anti-SSB**			**132/254 (52.0)**	**3/163 (1.8)**	**<0.001**
**Positive RF**			**119/201 (59.2)**	**8/155 (5.2)**	**<0.001**
**RF, IU/mL**			**76.9 ± 134.4 (201)**	**12.2 ± 32.4 (155)**	**<0.001**
**IgG, g/L**			**18.1 ± 8.8 (247)**	**10.9 ± 2.7 (164)**	**<0.001**
**IgM, g/L**			**1.3 ± 0.9 (247)**	**1.1 ± 0.7 (163)**	**0.007**
IgA, g/L			5.6 ± 42.6 (244)	2.4 ± 1.1 (164)	0.336
**C3, g/L**			**1.3 ± 0.3 (242)**	**1.3 ± 0.3 (159)**	**0.005**
**C4, g/L**			**0.2 ± 0.1 (241)**	**0.3 ± 0.1 (158)**	**<0.001**
Minor salivary gland biopsy features					
** Focus score**			**1.7 ± 1.0 (114)**	**0.3 ± 0.3 (96)**	**<0.001**
** Focus score ≥1**			**108/137 (78.8)**	**0/112 (0.0)**	**<0.001**
Atrophy	Mild		33/55 (60.0)	31/53 (58.5)	0.943
	Moderate		10/55 (18.2)	11/53 (20.8)	
	Severe		12/55 (21.8)	11/53 (20.8)	
Fibrosis	Mild		35/53 (66.0)	27/44 (61.4)	0.765
	Moderate		8/53 (15.1)	6/44 (13.6)	
	Severe		10/53 (18.9)	11/44 (25.0)	
Fat replacement			31/48 (64.6)	36/59 (61.0)	0.841
ESSPRI			5.9 ± 2.1 (178)	6.4 ± 2.1 (131)	0.062
**ESSDAI**			**4.7 ± 4.9 (257)**		
HAD anxiety			8.6 ± 4.6 (159)	9.0 ± 4.8 (113)	0.426
HAD depression			6.9 ± 4.0 (160)	7.5 ± 4.1 (114)	0.285
**EQ5D**			**0.6 ± 0.3 (161)**	**0.5 ± 0.3 (116)**	**0.007**
**Health state^a^**			**60.4 ± 21.6 (160)**	**53.5 ± 21.7 (116)**	**0.010**
**OSDI**			**42.6 ± 25.1 (158)**	**50.4 ± 25.3 (115)**	**0.012**
Comorbidities					
** Hypothyroidism**			**51/245 (20.8)**	**20/157 (12.7)**	**0.044**
Type 2 diabetes			16/245 (6.5)	17/158 (10.8)	0.140
** FM**			**34/241 (14.1)**	**38/154 (24.7)**	**0.011**

Variables presented as mean ± s.d. (*N*) or *n*/*N* (%), as adequate. ^a^Patient-reported assessment of current health on a 0–100 scale, where 100 indicates the best and 0 the worst imaginable health state. *n*: number of patients positive for the variable of interest; *N*: number of patients without missing information regarding the variable of interest; CC: classification criteria; ESSPRI: EULAR Sjögren’s Syndrome Patient Reported Index; ESSDAI: EULAR Sjögren’s Disease Activity Index. Significant values (*P* < 0.05) in bold.

Other differences were seen when comparing SjD patients with sicca. SjD patients were more frequently non-White compared with sicca (*P < *0.001). Additionally, hypothyroidism was more prevalent in the SjD population, whereas FM was more frequent within the sicca group (Table 1).

Among ever smokers, SjD patients had a lower smoking exposure, measured in pack-year units [10.8 ± 11.0 (*n* = 37) *vs* 15.3 ± 13.1 (*n* = 40), *P = *0.048]. The time between the onset of symptoms and smoking cessation was calculated for past smokers, revealing that the majority of SjD and sicca stopped smoking before symptom onset, with 71.8% and 67.6%, respectively, stopping >5 years before symptom onset.

When adding information on passive smoking, SjD patients were more frequently never exposed, i.e. non-smokers with no passive smoking history [31.2% (*n* = 49/157) *vs* 20.3% (*n* = 24/118), *P = *0.032]. However, amongst never smokers there was no statistically significant difference in passive smoking between SjD and sicca [46.7% (*n* = 43/92) *vs* 52.9% (*n* = 27/51), *P = *0.491].

There was a higher tendency for SjD population to report no alcohol consumption, although this did not reach statistical significance [44.0% (*n* = 81/184) *vs* 30.8% (*n* = 41/133), *P = *0.058].

In a multivariate analysis, after checking for collinearity (data not shown), both current smoking (OR 0.37, 95% CI 0.15–0.92, *P = *0.032) and White ethnicity (OR 0.26, 95% CI 0.12–0.56, *P = *0.001) were negatively associated with SjD diagnosis in suspected patients, independent of age and sex (Supplementary Table S1).

### Smoking exposure and SjD features

Amongst SjD patients meeting classification criteria (Table 2), IgG levels were significantly higher in never smokers (*P = *0.003), with a more prevalent active biological domain in ESSDAI at inclusion (*P = *0.038). Past smokers were more frequently of White ethnicity (*P = *0.003).

**Table 2:** Clinical and laboratorial characteristics in Sjögren’s disease patients according to smoking status.

Sjögren’s disease meeting 2016 CC		Never smoker (*n* = 155)	Current smoker (*n* = 13)	Past smoker (*n* = 52)	Univariate analysis (*P*-value)
**Age at inclusion, years**		**53.8 ± 15.0 (155)**	**48.2 ± 14.0 (13)**	**60.3 ± 12.9 (51)**	**0.005**
**Age at diagnosis, years**		**49.3 ± 15.9 (143)**	**43.7 ± 14.4 (13)**	**54.8 ± 11.8 (47)**	**0.028**
**Age at symptom onset, years**		**45.6 ± 15.6 (149)**	**39.7 ± 14.9 (13)**	**50.8 ± 12.9 (49)**	**0.029**
Symptom duration, years		8.4 ± 9.4 (149)	8.5 ± 8.9 (13)	9.1 ± 8.8 (50)	0.810
**Female sex**		**149/155 (96.1)**	**9/13 (69.2)**	**50/52 (96.2)**	**<0.001**
**Ethnicity**	**White**	**73/120 (60.8)**	**6/10 (60.0)**	**40/43 (93.0)**	**0.003**
	**Asian**	**33/120 (27.5)**	**4/10 (40.0)**	**0/43 (0.0)**	
	**Black**	**5/120 (4.2)**	**0/10 (0.0)**	**1/43 (2.3)**	
	**Other**	**9/120 (7.5)**	**0/10 (0.0)**	**2/43 (4.7)**	
BMI, kg/m^2^		27.6 ± 10.6 (149)	24.6 ± 5.8 (13)	28.2 ± 6.1 (51)	0.053
Clinical data					
Positive anti-SSA		137/155 (88.4)	11/13 (84.6)	44/52 (84.6)	0.746
Positive anti-SSB		81/152 (53.3)	4/13 (30.8)	25/52 (48.1)	0.270
Positive RF		73/119 (61.3)	5/10 (50.0)	17/39 (43.6)	0.138
RF, IU/mL		79.7 ± 120.6 (119)	145.6 ± 372.3 (10)	53.4 ± 92.5 (39)	0.141
** IgG, g/L**		**19.0 ± 9.3 (148)**	**14.0 ± 3.8 (12)**	**15.1 ± 6.7 (52)**	**0.003**
IgM, g/L		1.3 ± 0.8 (148)	1.4 ± 1.3 (12)	1.3 ± 1.4 (52)	0.174
IgA, g/L		3.0 ± 1.4 (147)	2.9 ± 1.1 (12)	15.6 ± 93.2 (51)	0.196
C3, g/L		1.2 ± 0.3 (149)	1.2 ± 0.3 (12)	1.3 ± 0.3 (46)	0.319
C4, g/L		0.2 ± 0.1 (149)	0.2 ± 0.1 (12)	0.2 ± 0.1 (46)	0.586
Minor salivary gland biopsy features					
Focus score		1.8 ± 0.9 (73)	1.6 ± 1.4 (6)	1.6 ± 1.0 (20)	0.742
Focus score ≥1		71/88 (80.7)	4/6 (66.7)	20/25 (80.0)	0.710
Atrophy	Mild	23/41 (56.1)	2/3 (66.7)	6/9 (66.7)	0.707
	Moderate	7/11 (17.1)	1/3 (33.3)	2/9 (22.2)	
	Severe	11/41 (26.8)	0/3 (0.0)	1/9 (11.1)	
Fibrosis	Mild	26/39 (66.7)	2/3 (66.7)	5/9 (55.6)	0.353
	Moderate	4/39 (10.3)	1/3 (33.3)	3/9 (33.3)	
	Severe	9/39 (10.3)	0/3 (0.0)	1/9 (11.1)	
Fat replacement		25/38 (65.8)	2/3 (66.7)	4/6 (66.7)	0.999
Germinal centres		18/70 (25.7)	2/6 (33.3)	2/19 (10.5)	0.315
Lymphoepithelial lesions		12/41 (29.3)	0/3 (0.0)	3/10 (30.0)	0.542
Schirmer’s test ≤5 mm/5 min		94/147 (63.9)	2/13 (15.4)	12/50 (24.0)	0.554
Unstimulated salivary flow ≤0.1 mL/min		97/146 (66.4)	8/12 (66.7)	29/49 (59.2)	0.648
Unstimulated salivary flow, mL/min		0.1 ± 0.1 (145)	0.1 ± 0.2 (12)	0.1 ± 0.1 (49)	0.756
Dental caries		41/134 (30.6)	3/10 (30.0)	10/45 (22.2)	0.558
**Tooth loss**		**67/135 (49.6)**	**3/11 (27.3)**	**32/47 (68.1)**	**0.020**
ESS eye dryness		5.7 ± 3.0 (48)	7.5 ± 2.1 (2)	6.1 ± 3.1 (12)	0.736
ESS mouth dryness		6.4 ± 3.0 (48)	7.0 ± 0.0 (2)	7.7 ± 3.1 (12)	0.252
ESSPRI dryness		6.2 ± 2.8 (124)	7.0 ± 0.9 (9)	6.9 ± 2.7 (45)	0.247
ESSPRI fatigue		5.9 ± 2.6 (122)	6.9 ± 2.1 (9)	6.6 ± 2.8 (45)	0.155
ESSPRI pain		5.1 ± 3.0 (123)	5.8 ± 3.2 (9)	5.7 ± 2.8 (45)	0.494
ESSPRI		5.7 ± 2.2 (124)	6.6 ± 1.7 (9)	6.4 ± 1.9 (45)	0.117
ESSDAI		4.5 ± 4.7 (155)	5.5 ± 5.0 (13)	5.2 ± 5.6 (52)	0.618
**Active biological domain in ESSDAI**		**86/155 (55.5)**	**4/13 (30.8)**	**20/52 (38.5)**	**0.038**
**HAD anxiety**		**7.8 ± 4.5 (111)**	**10.8 ± 5.4 (9)**	**10.1 ± 4.4 (39)**	**0.013**
HAD depression		6.5 ± 3.8 (111)	8.6 ± 4.8 (9)	7.9 ± 4.2 (40)	0.114
**EQ5D**		**0.7 ± 0.3 (111)**	**0.4 ± 0.3 (9)**	**0.6 ± 0.3 (41)**	**0.017**
Health state^a^		60.7 ± 23.2 (110)	56.2 ± 19.7 (9)	60.5 ± 17.2 (41)	0.628
OSDI		41.9 ± 24.8 (111)	47.1 ± 24.8 (8)	47.1 ± 31.8 (39)	0.846

Variables presented as mean ± s.d. years (*N*) or *n*/*N* (%), as adequate. ^a^Patient-reported assessment of current health on a 0–100 scale, where 100 indicates the best and 0 the worst imaginable health state. *n*: number of patients positive for the variable of interest; *N*: number of patients without missing information regarding the variable of interest; CC: classification criteria; ESSPRI: EULAR Sjögren’s Syndrome Patient Reported Index; ESSDAI: EULAR Sjögren’s Disease Activity Index; OSDI: Ocular Surface Disease Index. Significant values (*P* < 0.05) in bold.

When dividing SjD patients meeting classification criteria into non-exposed, passive smokers and ever smokers (Table 3), never smokers with no history of passive smoking exhibited significantly higher levels of RF (*P = *0.038), IgG (*P = *0.004) and IgM (*P = *0.006). While∼20% of the SjD cohort were receiving HCQ and 5% other DMARDs, the proportion of treated patients did not differ according to smoking status (data not shown).

**Table 3:** Sjogren’s disease population characteristics divided according to smoking status as non-exposed/passive smoker/ever smoker.

Sjögren’s disease meeting 2016 CC	Non-exposed (*n* = 49)	Passive smoker (*n* = 43)	Ever smoker (*n* = 65)	Univariate analysis (*P*-value)
Positive RF	25/36 (69.4)	16/31 (51.6)	22/49 (44.9)	0.076
**RF, IU/mL**	**117.8 ± 151.4 (36)**	**58.3 ± 119.0 (31)**	**72.2 ± 184.8 (49)**	**0.038**
**IgG, g/L**	**21.0 ± 11.5 (47)**	**15.5 ± 5.9 (43)**	**14.9 ± 6.3 (64)**	**0.004**
**IgM, g/L**	**1.6 ± 0.9 (47)**	**1.2 ± 0.7 (43)**	**1.3 ± 1.3 (64)**	**0.006**
ESS mouth dryness	6.0 ± 3.4 (13)	7.3 ± 2.4 (15)	7.6 ± 2.9 (14)	0.321
ESS eye dryness	5.6 ± 3.4 (13)	6.2 ± 2.8 (15)	6.3 ± 2.9 (14)	0.984
**ESSPRI fatigue**	**5.6 ± 2.5 (47)**	**5.8 ± 2.8 (41)**	**6.7 ± 2.7 (54)**	**0.040**
ESSPRI pain	4.6 ± 3.0 (48)	5.4 ± 2.9 (41)	5.7 ± 2.8 (54)	0.143
ESSPRI dryness	6.0 ± 2.7 (48)	6.4 ± 2.6 (42)	6.9 ± 2.5 (54)	0.218
**ESSPRI**	**5.4 ± 2.2 (48)**	**5.9 ± 2.1 (42)**	**6.4 ± 1.9 (54)**	**0.039**
**HAD anxiety**	**8.2 ± 4.4 (49)**	**6.8 ± 3.5 (43)**	**10.3 ± 4.6 (49)**	**0.001**
HAD depression	6.0 ± 3.8 (48)	6.7 ± 3.4 (43)	8.0 ± 4.3 (49)	0.077
**EQ5D**	**0.8 ± 0.2 (48)**	**0.7 ± 0.2 (43)**	**0.5 ± 0.3 (50)**	**<0.001**

Variables presented as mean ± s.d. years (*N*) or *n*/*N* (%), as adequate. *n*: number of patients positive for the variable of interest, *N*: number of patients without missing information regarding the variable of interest; CC: classification criteria; ESSPRI: EULAR Sjögren’s Syndrome Patient Reported Index; ESSDAI: EULAR Sjögren’s Disease Activity Index. Significant values (*P* < 0.05) in bold.

A linear regression model including age, sex, ethnicity and classification (SjD meeting 2016 classification criteria *vs* sicca) confirmed that never smoking (β 1.94, 95% CI 0.12–3.77, *P = *0.037) remained a significant predictor of IgG levels [R^2^ = 0.287, F(5, 254) = 20.487, *P < *0.001, Supplementary Table S2]. When looking at the entire OASIS population (both SjD and sicca), an inverse correlation trend was observed between smoking exposure (measured in pack-years) and IgG levels (rho = −0.186, *P = *0.076), although this did not reach statistical significance.

Considering a wider population of clinically diagnosed SjD patients, regardless of classification criteria (Supplementary Table S3), those with a history of smoking exposure (including passive smoking) were less frequently positive for anti-Ro antibodies (*P = *0.008) and RF (*P = *0.016), and had significantly lower IgG and RF levels (*P = *0.002 and *P = *0.006, respectively).

Interestingly, looking at our sicca cohort, IgG levels were also higher in never smokers (*P = *0.001, Table 4).

**Table 4:** Clinical and laboratorial characteristics in sicca individuals according to smoking status.

Sicca		Never smoker (*n* = 76)	Current smoker (*n* = 18)	Past smoker (*n* = 49)	Univariate analysis (*P*-value)
Age at inclusion, years		56.2 ± 12.3 (75)	53.3 ± 12.4 (18)	56.2 ± 9.5 (48)	0.598
Age at symptom onset, years		48.6 ± 14.8 (68)	48.8 ± 11.1 (15)	48.3 ± 11.5 (47)	0.989
Symptom duration, years		7.6 ± 8.7 (69)	5.0 ± 2.0 (15)	8.1 ± 6.7 (48)	0.372
Female sex		68/76 (89.5)	18/18 (100.0)	44/49 (89.8)	0.356
Ethnicity	White	43/51 (84.3)	12/13 (92.3)	31/32 (96.9)	0.073
	Asian	7/51 (13.7)	0/13 (0.0)	1/32 (3.1)	
	Black	1/51 (2.0)	0/13 (0.0)	0/32 (0.0)	
	Other	0/51 (0.0)	1/13 (7.7)	0/32 (0.0)	
**BMI, kg/m^2^**		**27.2 ± 6.1 (74)**	**26.1 ± 4.8 (18)**	**31.7 ± 7.7 (45)**	**<0.001**
Clinical data					
Positive RF		3/69 (4.3)	2/15 (13.3)	2/46 (4.3)	0.349
RF, IU/mL		14.0 ± 46.1 (69)	14.7 ± 20.5 (15)	9.5 ± 5.0 (46)	0.769
** IgG, g/L**		**11.6 ± 2.7 (74)**	**9.2 ± 2.5 (17)**	**10.4 ± 2.6 (47)**	**0.001**
IgM, g/L		1.1 ± 0.8 (73)	1.1 ± 0.4 (17)	1.1 ± 0.6 (47)	0.939
IgA, g/L		2.5 ± 1.1 (74)	2.2 ± 0.8 (17)	2.3 ± 0.9 (47)	0.261
C3, g/L		1.3 ± 0.3 (73)	1.4 ± 0.3 (17)	1.4 ± 0.3 (44)	0.263
C4, g/L		0.3 ± 0.1 (72)	0.3 ± 0.1 (17)	0.3 ± 0.1 (44)	0.254
Schirmer’s test ≤5 mm/5 min		29/71 (40.8)	7/17 (41.2)	18/47 (38.3)	0.957
Unstimulated salivary flow ≤0.1 mL/min		35/71 (49.3)	7/18 (38.9)	25/49 (51.0)	0.668
Unstimulated salivary flow, mL/min		0.2 ± 0.3 (69)	0.2 ± 0.3 (17)	0.1 ± 0.1 (49)	0.159
Dental caries		11/65 (16.9)	4/16 (25.0)	7/46 (15.2)	0.668
Tooth loss		22/63 (34.9)	8/16 (50.0)	19/45 (42.2)	0.489
ESS eye dryness		7.5 ± 2.2 (20)	6.6 ± 2.2 (5)	7.7 ± 1.3 (20)	0.529
ESS mouth dryness		7.0 ± 2.6 (19)	6.0 ± 1.2 (5)	6.9 ± 2.0 (20)	0.675
ESSPRI dryness		7.0 ± 1.9 (68)	7.2 ± 2.0 (14)	6.8 ± 2.1 (47)	0.793
**ESSPRI fatigue**		**6.0 ± 2.7 (68)**	**6.4 ± 3.1 (14)**	**7.3 ± 2.6 (48)**	**0.048**
ESSPRI pain		5.2 ± 2.8 (67)	5.7 ± 2.8 (14)	6.4 ± 2.6 (47)	0.065
ESSPRI		6.0 ± 2.0 (68)	6.4 ± 2.3 (14)	6.9 ± 2.1 (48)	0.117
HAD anxiety		8.6 ± 5.3 (58)	9.4 ± 5.0 (12)	9.5 ± 4.1 (43)	0.602
HAD depression		6.9 ± 4.1 (59)	7.8 ± 3.9 (12)	8.2 ± 4.0 (43)	0.291
EQ5D		0.6 ± 0.3 (60)	0.5 ± 0.3 (12)	0.5 ± 0.3 (44)	0.378
**Health state^a^**		**58.9 ± 20.9 (60)**	**48.1 ± 24.2 (12)**	**47.8 ± 20.6 (44)**	**0.022**
OSDI		48.4 ± 25.9 (59)	50.0 ± 25.8 (12)	53.1 ± 24.7 (44)	0.648

Variables presented as mean ± s.d. years (*N*) or *n*/*N* (%), as adequate. ^a^Patient-reported assessment of current health on a 0–100 scale, where 100 indicates the best and 0 the worst imaginable health state. *n*: number of patients positive for the variable of interest, *N*: number of patients without missing information regarding the variable of interest; ESSPRI: EULAR Sjögren’s Syndrome Patient Reported Index; ESSDAI: EULAR Sjögren’s Disease Activity Index. Significant values (*P* < 0.05) in bold.

No differences were observed in the main features of minor salivary gland biopsies that could be attributed to smoking status within our cohort.

### Symptom severity and smoking

In patients meeting the 2016 ACR/EULAR SjD criteria, ESSPRI scores were higher in ever smokers compared with non-exposed (*P = *0.039), with fatigue being a significant contributing factor to this difference (*P = *0.040, Table 3). Similarly, in sicca, symptoms of fatigue, but not dryness, were significantly higher in ever smokers (*P = *0.048, Table 4).

### Comparison with matched healthy controls

Within the population-based control data, 17 081 had complete smoking status data with 61.1% never smokers, 24.8% past and 14.2% current smokers, and 17 180 had binary smoking status data (ever/never smoker), with 60.7% never smokers. The case populations were matched to the controls in terms of sex, age and ethnicity (White *vs* non-White). From the aforementioned total, 576 healthy controls were matched to 192 SjD patients (Table 5), and 345 matched to 115 sicca individuals (Table 6), with loss of sicca and SjD cases mostly due to availability of matches and, rarely, due to missing ethnicity data.

**Table 5:** Demographic and smoking data between SjD patients and matched population controls.

		SjD meeting 2016 CC (*n* = 192)	Matched population controls (*n* = 576)	Univariate analysis (*P*-value)
Age at inclusion, years		54.7 ± 14.8 (192)	54.7 ± 14.7 (576)	0.980
Female sex		182/192 (94.8)	546/576 (94.8)	1.000
Ethnicity	White	130/192 (67.7)	390/576 (67.7)	1.000
	Non-White	62/192 (32.3)	186/576 (32.3)	
Smoking status	Never smoker	133/192 (69.3)	414/576 (71.9)	0.260
	Current smoker	12/192 (6.3)	49/576 (8.5)	
	Past smoker	47/192 (24.5)	113/576 (19.6)	
Smoking status	Never smoker	133/192 (69.3)	414/576 (71.9)	0.490
	Ever smoker (current or past)	59/192 (30.7)	162/576 (28.1)	

Variables presented as mean ± s.d. (*N*) or *n*/*N* (%), as adequate. *n*: number of patients positive for the variable of interest; *N*: number of patients without missing information regarding the variable of interest; CC: classification criteria.

**Table 6:** Demographic and smoking data between sicca individuals and matched population controls.

		Sicca (*n* = 115)	Matched population controls (*n* = 345)	Univariate analysis (*P*-value)
Age at inclusion, years		56.4 ± 11.4 (115)	56.4 ± 11.4 (345)	0.986
Female sex		105/115 (91.3)	315/345 (91.3)	1.000
Ethnicity	White	102/115 (88.7)	306/345 (88.7)	1.000
	Non-White	13/115 (11.3)	39/345 (11.3)	
Smoking status	Never smoker	58/115 (50.4)	210/345 (60.9)	0.082
	Current smoker	14/115 (12.2)	43/345 (12.5)	
	Past smoker	43/115 (37.4)	92/345 (26.7)	
**Smoking status**	**Never smoker**	**58/115 (50.4)**	**210/345 (60.9)**	**0.049**
	**Ever smoker (current or past)**	**57/115 (49.6)**	**135/345 (39.1)**	

Variables presented as mean ± s.d. (*N*) or *n*/*N* (%), as adequate. *n*: number of patients positive for the variable of interest; *N*: number of patients without missing information regarding the variable of interest. Significant values (*P* < 0.05) in bold.

When compared with the matched healthy APS control population, no differences were seen in the smoking patterns of our SjD cohort (Table 5). Sicca individuals, on the other hand, had statistically significantly higher rates of ever smoking than the matched general population [49.6% (*n* = 57/115) *vs* 39.1% (*n* = 135/345), *P = *0.049, Table 6]. Whilst the sicca cohort had higher rates of past smoking than the control group, these results did not reach statistical significance (*P = *0.082).

In the full regression models using the entire APS control population, ever smoking was significantly associated with higher odds of a sicca diagnosis (OR 1.75, 95% CI 1.20–2.55, *P = *0.004, Supplementary Table S4), following adjustment for age, sex and ethnicity. When smoking status was subdivided, past smoking remained significant (OR 2.04, 95% CI 1.36–3.08, *P < *0.001), whereas current smoking was not (OR 1.30, 95% CI 0.72–2.35, *P* = 0.391, Supplementary Table S5). Conversely, no association was seen between smoking and SjD diagnosis in adjusted analyses (Supplementary Tables S6 and S7).

## Discussion

The relationship between smoking and SjD has been complex to disentangle. Our findings comparing SjD and sicca support the notion that current smoking is inversely related to SjD, aligning with existing literature. This protective role is further supported by the multivariate analysis, where both current smoking and White ethnicity were independently associated with a reduced likelihood of an SjD diagnosis in suspected patients. Importantly, our results also demonstrate a negative association between SjD and past smoking, an area in which existing evidence has been inconsistent.

The mechanisms underpinning this apparent protective effect remain unclear. Although the hypothesis of reverse causation has been commonly invoked, whereby patients stop smoking due to early dryness symptoms, our study shows that the majority of past smokers stopped smoking long before symptom onset, suggesting that smoking cessation is not merely a response to early disease manifestation. Interestingly, in our study, ESSPRI was higher in ever smokers compared with non-exposed patients. This finding highlights the broader impact of smoking on patient-reported outcomes and quality of life. However, this was true for fatigue and not dryness. Similarly, in patients with sicca, fatigue, but not dryness, was also more severe among ever smokers, suggesting that smoking may contribute to fatigue severity regardless of the underlying diagnosis, and not showing a clear association between smoking and dryness in either population. Notably, previous studies have also failed to demonstrate a consistent association between smoking and ocular [15–17] or oral dryness [18] or even overall ESSPRI scores [19], implying that smoking does not directly worsen sicca symptoms.

Furthermore, when we compared the SjD and sicca groups to population controls, we found that it was the sicca group which differed, being associated with a higher risk of smoking. Whilst this may suggest that smoking has no relationship to SjD, the association between smoking and lower immunoglobulins, a biomarker of B-cell hyperactivity in SjD, is striking.

Looking at clinically diagnosed SjD patients, which allowed for the inclusion of more patients who were anti-Ro/SSA or biopsy negative, smoking was associated with a lower frequency of positive anti-Ro/SSA and RF as well as lower RF levels. Similarly, other studies have found fewer smokers amongst anti-Ro/SSA positive patients [5, 6, 8, 10, 20]. Some have analysed this association in a mixed cohort of both patients and controls allowing for the confounding factor of a possible negative association between smoking and the diagnosis itself. Data on RF have been less consistent. Whereas smoking promotes autoantibody formation in RA [21, 22], our data indicate the opposite pattern in SjD, suggesting disease-specific immunomodulatory effects. The lower immunoglobulin levels among smokers, also observed in the sicca group, reinforce the notion that smoking may suppress B-cell activation and antibody production. These data are consistent with literature suggesting that smoking can suppress immunoglobulin levels in other populations [23]. Together, these results suggest a hypothesis that smoking dampens systemic B cell–mediated immune activation and modulates the phenotype of glandular immunopathology that arises following dysregulation of salivary gland epithelium.

In accordance with available literature, our study found no significant differences in most systemic disease domains accounted for in the ESSDAI [24, 25], with the exception of the biological domain which may be expected, considering the influence of smoking on IgG levels in our cohort [26]. These findings were independent of therapeutic drug exposure, as the proportion of patients receiving HCQ or other DMARDs did not differ by smoking status, making it unlikely that treatment confounded the observed relationship between smoking and IgG levels. This further supports an immunoregulatory effect of smoking on humoral activation.

In our study, however, we did not detect differences in salivary gland histopathology across smoking categories. This result, contrasting with previous literature [6, 10, 20, 24, 26], may reflect the limited sample size in our own and some preceding studies or, on the other hand, that glandular damage may have mechanisms independent of systemic immunological activity.

Data on passive smoking and SjD are sparse, but our study provide some insights. We observed that SjD patients were more frequently non-exposed to passive smoking compared with sicca. This finding contrasts with the single existing study by Karabulut *et al.* [3], which found a positive correlation between passive smoking and SjD. The discrepancy may stem from differences in study populations, methodologies or environmental factors. Passive smoking data are, nevertheless, a strength in our study, as passive smoke exposure could represent a meaningful confounder when studying never smokers, and ‘non-exposed’ individuals may provide a purer comparator group in future studies.

The divergent smoking patterns between SjD patients and sicca also underscore the importance of control group selection in SjD research. Non-Sjögren’s sicca syndrome is a clinically heterogeneous condition with incompletely understood pathophysiology. In our previous proteomic work [27], a subgroup of sicca individuals was identified with a metabolic signature that correlated with higher BMI and symptom burden. Our current findings, linking sicca to higher smoking prevalence, further support the concept of a metabolically influenced sicca phenotype, where lifestyle and metabolic factors, including smoking, obesity and systemic inflammation, contribute to dryness and fatigue symptoms without overt autoimmunity. This distinction may have implications for the design of future case-control studies and for understanding disease mechanisms underlying different sicca subtypes.

Our study has several limitations. Its cross-sectional and monocentric design limits causal inference and generalizability, and smoking data were self-reported, introducing potential recall bias. Nevertheless, the inclusion of passive smoking data, rigorous phenotyping, and the use of both disease and population-based control groups are notable strengths. To our knowledge, only one previous study has incorporated both healthy and dryness controls [28], but recruited dry-eye syndrome patients with no minor salivary gland biopsy in the dryness group, which could possibly include undiagnosed SjD patients.

Furthermore, while the direct biological effects of tobacco on immune function and overall health are well established, smoking behaviours often coexist with other lifestyle and environmental factors [29] (such as alcohol consumption, sedentary behaviour and dietary patterns). These clustered exposures can exert cumulative influences on immune markers and on the risk and presentation of SjD [11, 30]. Although this raises the possibility that some of the observed associations may reflect a broader behavioural or environmental profile rather than smoking alone—a limitation common to all studies on health behaviours—BMI and alcohol consumption data in our cohort did not appear to influence SjD diagnosis or presentation. Taken together with data on smoking exposure in pack-years and the influence of smoking on IgG levels, our findings support a genuine biological contribution of smoking to the observed phenotype.

In conclusion, our study demonstrates that both current and past smoking are negatively associated with SjD compared with sicca, independent of demographic factors. Among SjD patients, smoking exposure is linked to lower immunoglobulin levels and reduced seropositivity, supporting a biologically plausible immunomodulatory effect that shapes the pathology arising following salivary gland insult. Alternatively, smoking may predispose to a non-autoimmune, metabolically influenced sicca phenotype rather than exerting a true protective effect against SjD. Future multicentric and longitudinal studies integrating metabolic, immunologic and environmental factors are warranted to further elucidate the interplay between smoking, autoimmunity and mucosal dryness.

## Supplementary Material

keag334_Supplementary_Data

## Data Availability

The data underlying this article will be shared on reasonable request to the corresponding author.
