# Supplementary material for: Impact of smoking status on Sjögren’s disease diagnosis and phenotype: results from a cohort study with matched population controls
Source: Rheumatology (Oxford). 2026 Jun 24;65(7):keag334. doi: 10.1093/rheumatology/keag334 (PMC13384511; doi:10.1093/rheumatology/keag334)
Supplement: keag334_Supplementary_Data [file keag334_supplementary_data.docx]

**Supplementary material:**

Supplementary Table S1 - Multivariate analysis for predictors of Sjogren’s disease diagnosis compared to sicca patients

| Variables | | OR | 95% CI | p-value |
| --- | --- | --- | --- | --- |
| Age at inclusion | | 1.003 | 0.982-1.024 | 0.807 |
| Female sex | | 2.118 | 0.791-5.672 | 0.136 |
| **White ethnicity** | | **0.256** | **0.117-0.563** | **0.001** |
| **Smoking status**  **(vs never smoking)** | **Current smoking** | **0.367** | **0.146-0.920** | **0.032** |
|  | Past smoking | 0.787 | 0.433-1.428 | 0.431 |
| Abbreviations: OR – odds ratio; CI – confidence interval | | | | |

Supplementary Table S2 - Multivariate linear regression for predictors of IgG levels

| Variables | β | 95% CI | p-value |
| --- | --- | --- | --- |
| Age at inclusion | 0.002 | -0.065 – 0.068 | 0.956 |
| Female sex | 0.199 | -3.062 – 3.461 | 0.904 |
| **White ethnicity** | **-6.049** | **-8.217 – -3.881** | **<0.001** |
| **Never smoking (vs ever smoking)** | **1.941** | **0.113 – 3.768** | **0.037** |
| **Sjögren’s disease* (vs sicca)** | **5.222** | **3.413 – 7.031** | **<0.001** |
| Abbreviations: CI – confidence interval; *meeting 2016 ACR/EULAR classification criteria | | | |

Supplementary Table S3 - Clinical and laboratorial characteristics in patients with a clinical diagnosis of Sjögren’s according to smoking status including passive smoking information

| **Clinical diagnosis of Sjögren’s disease** | **Non-exposed (n=53)** | **Passive smoker (n=54)** | **Ever smoker (n=85)** | **Univariate analysis (p-value)** |
| --- | --- | --- | --- | --- |
| Classification criteria | | | | |
| 2002 AECG CC | 47/53 (88.7) | 44/54 (81.5) | 60/83 (72.3) | 0.064 |
| 2016 ACR/EULAR CC | 49/53 (92.5) | 43/54 (79.6) | 65/83 (78.3) | 0.083 |
| Clinical data | | | | |
|  | | | | |
| **Positive anti-SSA** | **49/53 (92.5)** | **37/54 (68.5)** | **67/85 (78.8)** | **0.008** |
| Positive anti-SSB | 31/53 (58.5) | 22/53 (41.5) | 34/85 (40.0) | 0.083 |
| **Positive rheumatoid factor** | **26/38 (68.4)** | **16/38 (42.1)** | **26/64 (40.6)** | **0.016** |
| **Rheumatoid factor, IU/mL** | **126.7±165.6 (38)** | **48.9±109.0 (38)** | **61.4±164.0 (64)** | **0.006** |
| **IgG, g/L** | **20.9±11.2 (51)** | **15.0±5.5 (53)** | **15.8±9.0 (84)** | **0.002** |
| Minor salivary gland biopsy features | | | | |
| Focus score | 1.9±0.9 (20) | 1.6±1.1 (24) | 1.5±1.0 (32) | 0.379 |
| Focus score≥1 | 21/24 (87.5) | 22/32 (68.8) | 25/37 (67.6) | 0.181 |
|  | | | | |
| Schirmer’s test ≤ 5mm/5min | 33/52 (63.5) | 30/52 (57.7) | 48/82 (58.5) | 0.803 |
| Schirmer’s test = 0mm/5min | 21/52 (40.4) | 15/51 (29.4) | 22/81 (27.2) | 0.258 |
| Unstimulated salivary flow ≤ 0.1mL/min | 31/51 (60.8) | 34/51 (66.7) | 42/80 (52.5) | 0.260 |
| Unstimulated salivary flow = 0mL/min | 11/51 (21.6) | 14/51 (27.5) | 12/80 (15.0) | 0.218 |
| Unstimulated salivary flow, mL/min | 0.1±0.2 (50) | 0.1±0.2 (50) | 0.2±0.2 (80) | 0.092 |
| Variables presented as mean ± SD years (N) or n/N (%), as adequate; Abbreviations: n – number of patients positive for the variable of interest, N – number of patients without missing information regarding the variable of interest, SD – standard deviation; classification criteria | | | | |

Supplementary Table S4 - Multivariate analysis model for sicca (n=115) compared to APS healthy controls (n=17173) with dichotomised smoking status

| Variables | | OR | 95% CI | p-value |
| --- | --- | --- | --- | --- |
| **Age at inclusion** | | **1.012** | **1.002-1.023** | **0.020** |
| **Female sex** | | **10.309** | **5.348-19.608** | **<0.001** |
| White ethnicity | | 0.001 | 0.001-1.754 | 0.894 |
| **Smoking status**  **(vs never smoking)** | **Ever smoking** | **1.751** | **1.201-2.554** | **0.004** |
| Abbreviations: OR – odds ratio; CI – confidence interval | | | | |

Supplementary Table S5 - Multivariate analysis model for sicca (n=115) compared to APS healthy controls (n=17074) with complete smoking status data

| Variables | | OR | 95% CI | p-value |
| --- | --- | --- | --- | --- |
| **Age at inclusion** | | **1.012** | **1.001-1.022** | **0.032** |
| **Female sex** | | **10.309** | **5.376-19.608** | **<0.001** |
| White ethnicity | | 0.001 | 0.001-1.751 | 0.890 |
| **Smoking status**  **(vs never smoking)** | Current smoking | 1.297 | 0.716-2.347 | 0.391 |
|  | **Past smoking** | **2.042** | **1.356-3.077** | **<0.001** |
| Abbreviations: OR – odds ratio; CI – confidence interval | | | | |

Supplementary Table S6 - Multivariate analysis model for SjD (n=192) compared to APS healthy controls (n=17173) with dichotomised smoking status

| Variables | | OR | 95% CI | p-value |
| --- | --- | --- | --- | --- |
| **Age at inclusion** | | **1.015** | **1.007-1.023** | **<0.001** |
| **Female sex** | | **16.393** | **8.696-31.250** | **<0.001** |
| **White ethnicity** | | **0.0003** | **0.0002-0.0004** | **<0.001** |
| Smoking status  (vs never smoking) | Ever smoking | 1.012 | 0.732-1.397 | 0.945 |
| Abbreviations: OR – odds ratio; CI – confidence interval | | | | |

Supplementary Table S7 - Multivariate analysis model for SjD (n=192) compared to APS healthy controls (n=17074) with complete smoking status data

| Variables | | OR | 95% CI | p-value |
| --- | --- | --- | --- | --- |
| **Age at inclusion** | | **1.014** | **1.006-1.023** | **<0.001** |
| **Female sex** | | **16.393** | **8.621-31.250** | **<0.001** |
| **White ethnicity** | | **0.0003** | **0.0002-0.0004** | **<0.001** |
| Smoking status  (vs never smoking) | Current smoking | 0.613 | 0.336-1.119 | 0.111 |
|  | Past smoking | 1.257 | 0.883-1.788 | 0.204 |
| Abbreviations: OR – odds ratio; CI – confidence interval | | | | |
